# Synergistic Effect of Metal Oxide and Carbon Nanoparticles on the Thermal and Mechanical Properties of Polyimide Composite Films

**DOI:** 10.3390/polym15102298

**Published:** 2023-05-13

**Authors:** Alexandra L. Nikolaeva, Alexander N. Bugrov, Maria P. Sokolova, Igor V. Kuntsman, Elena N. Vlasova, Elena M. Ivan’kova, Ivan V. Abalov, Iosif V. Gofman

**Affiliations:** 1 Institute of Macromolecular Compounds, Russian Academy of Sciences, 199004 St. Petersburg, Russia; anbugrov@etu.ru (A.N.B.); pmarip@mail.ru (M.P.S.); i.v.kuntsman@gmail.com (I.V.K.); spectra@imc.macro.ru (E.N.V.); ivelen@mail.ru (E.M.I.); i.abalf@yandex.ru (I.V.A.); gofman@imc.macro.ru (I.V.G.); 2 Department of Physical Chemistry, Saint Petersburg Electrotechnical University (ETU “LETI”), ul. Professora Popova 5, 197022 St. Petersburg, Russia

**Keywords:** nanocomposites, polyimides, metal oxide nanoparticles, nanocarbon, binary filler, synergism

## Abstract

In this paper, we report on novel polyimide (PI) nanocomposites filled with binary mixtures of metal oxide (either TiO_2_ or ZrO_2_) nanoparticles and nanocarbon (either carbon nanofibers (CNFs) or functionalized carbon nanotubes (CNT_f_s)). The structure and morphology of the materials obtained were comprehensively studied. An exhaustive investigation of their thermal and mechanical properties was performed. We revealed a synergistic effect of the nanoconstituents with regard to a number of functional characteristics of the PIs compared with single-filler nanocomposites, including thermal stability, stiffness (below and above glass transition temperature), yield point, and temperature of flowing. Moreover, the possibility of manipulating the properties of the materials by choosing a proper combination of the nanofillers was demonstrated. The results obtained can become a platform in the design of PI-based engineering materials with tailored characteristics capable of operating in extreme conditions.

## 1. Introduction

Over the years, polyimides (PIs) being excellent engineering materials, have widely been used in many fields of industry, e.g., aerospace, mechanical and chemical industries, microelectronics, as well as production of fuel cells and household appliances. Such a variety of applications of PIs is ensured by a great set of their outstanding characteristics, including superb thermal resistance and thermal stability, high mechanical strength and good insulating properties, superior chemical and radiation resistance, etc. [1,2,3,4,5]. However, the further development of science and technology demands more materials that can operate well under harsh conditions. To fulfil the increasing need for the high-performance materials, PI-based nanocomposites have been vigorously investigated [6,7,8,9,10,11].

Metal oxide (MO) nanoparticles is a huge class of nanofillers having received a great attention because of the superiority of the MO-containing polymer nanocomposites over unfilled materials [12,13,14,15]. The widespread use of metal oxides comes also from the possibility of tuning the physico-chemical and working properties of the nanocomposites by changing the synthetic techniques of the nanocomponents, since the synthesis conditions determine their size, morphology, surface stoichiometry (the number of oxygen vacancies), and functionality [15,16,17]. The good prospects of PI-based materials filled with a number of MO nanospecies, such as ZrO_2_, TiO_2_, NiO, CeO_2_, and ZnO are reported in the literature [18,19,20,21,22,23,24,25,26,27,28].

The concept of filling PIs with one-dimensional (1D) carbon nanoparticles, nanofibers (CNF), and nanotubes (CNT) stems from the excellent reinforcing properties of these nanospecies with a high axial ratio (10^2^–10^3^) [29,30,31,32,33,34]. It is well-known that they are capable of forming net structures within polymer matrices and taking over a part of mechanical load applied to a composite material. As a result, a significant improvement in rigidity and strength of the material is observed. For example, the break stress of a nanocomposite based on PI filled with CNF can surpass that of the unfilled matrix by ~40% [32]. In [35], the authors report on an increase in Young’s modulus of a PI material resulted from filling the matrix with CNT by ~40% as well. The impact of carbon nanoparticles on thermal properties of PIs is generally less remarkable. The insertion of CNT as well as CNF in the PIs has been shown to change the glass transition temperature of the polymers only slightly [36,37,38], their thermal stability indices being rather unaffected.

The matrices filled with only one type of nanoparticles, either MO or carbon species, may often acquire only one enhanced working characteristic (be it rigidity, strength, thermal stability, etc.), while other properties are deteriorated. In this regard, it seems quite expedient to combine the said types of nanoparticles to tackle the issue of simultaneous and controlled manipulation of a wide range of operating properties of PI nanocomposites. In recent years, novel composite polymer materials filled with binary and even ternary mixtures of nanoparticles have been worked out, spanning from combinations of carbon nanoparticles of various morphologies [39,40,41,42,43] to those of cellulose nanoparticles and clay [44]. The synergism of the components regarding a number of properties of polymers has been demonstrated. Nanocomposites containing mixed carbon black/nanoclay, SiO_2_/CNT, and graphene/nanoclay fillers have been shown to outperform both the corresponding polymer matrices and nanocomposites with only single filler when considering their mechanical properties [45,46,47]. The synergistic effect of the constituents of the nanoclay/CNT combination regarding thermal stability of the resulting nanocomposite material was reported in [48].

Concerning PI-based nanocomposites, one should note a relative lack of studies on the systems filled with two types of nanoparticles. However, a steady growth in the number of publications on this topic has been observed over a span of ten years, indicating the relevance of the investigation of such ternary systems and prospects for their use in various fields. A positive influence of binary fillers, Al_2_O_3_ microspheres/boron nitride nanosheets, and aluminum nitride/boron nitride, on thermal conductivity of PIs has been registered in [49,50]. The authors suggested using these materials in microelectronics and thermal interface production. Yet, mechanical properties of the nanocomposites are worse than those of the corresponding matrices. As a result, the materials may fall short of the requirements of certain applications. PIs filled with two components, MO (either WO_3_ or PbO) nanoparticles and graphene, have been proven to possess electrical conductivity higher than that of the nanocomposites with a single type of nanofiller [51]. Even so, the capabilities of these nanocomposites from a practical viewpoint may well appear to be debatable due to the scarcity of information on their thermal and mechanical characteristics. Li et al. [52] discovered the synergism of the graphene nanoplatelets (GNP)/BN mixture with respect to thermal conductivity and dielectric properties of PIs, while the improvement of mechanical properties of PI films brought about by the same combination of fillers was demonstrated in [53]. We recently developed new PI-based composite film materials with binary nanoadditives, either CeO_2_/CNF or CeO_2_/carbon nanocones [54]. CeO_2_ nanospecies solely were shown to enhance thermal stability of the composites by ~30 °C, but the mechanical performance of the materials decreased compared with the host polymer. However, the insertion of the mixed nanosized MO/nanocarbon filler facilitated a simultaneous increase in thermal stability (by ~20 °C) and rigidity (by 15%) of the films. This revealed the synergism of the nanocomponents germane to operational properties of PI films.

Overall, the necessity in the comprehensive investigation of the impact of binary fillers consisting of nanosized metal oxides and carbon nanoparticles on a number of properties of PIs crucial for practical application is quite obvious. PIs being one of the foremost engineering polymers, their thermal and mechanical characteristics are of great interest. The modification of PI matrices with mixed fillers synergistically affecting a set of characteristics may well become a perspective tool in developing novel materials relevant to industrial usage.

In the present work, we address the problem of the development of advanced PI-based nanocomposites filled with binary mixtures of nanofillers, the latter being the combinations of either TiO_2_ or ZrO_2_ and either CNT or CNF. The following prerequisites determined the choice of the components: Both the oxides are non-toxic, low-cost, and widely used because they are able to augment the corrosion resistance of materials, possess a high dielectric constant, and can be used as pigment pieces for thermoregulating coatings in aerospace [55]. They are also characterized by high thermal and chemical stability. Moreover, these nanoparticles have demonstrated good application prospects in improving the functional properties of PIs [19,21,22,24,56,57]. We already discussed how the type, size, surface functionality, and concentration of these nanofillers can affect the thermal and mechanical properties of PI-based nanocomposites [58]. On the other hand, both CNT and CNF have long been used as reinforcing agents in PIs imparting greater rigidity and mechanical strength to the host polymers [59,60,61]. In this study we reveal the synergism of the aforementioned types of nanofillers in the context of simultaneous enhancement of key thermal and mechanical characteristics of PIs. We also investigate the structure and morphology of the nanocomposites developed. 

## 2. Materials and Methods

### 2.1. Materials

To obtain composite films with metal oxide and carbon nanoparticles, we used 15% solutions of polyamic acids (PAA) in N-methylpyrrolidone (NMP) based on pyromellitic dianhydride (PMDA) and 4,4′-oxydianilin (ODA), as well as on 1,3-bis(3,4-dicarboxyphenoxy)benzene dianhydride (dianhydride R) and 4,4′-bis(4″-aminophenoxy)biphenyl sulfone (diamine BAPS). Poly(pyromellitic dianhydride-co-4,4′-oxydianiline) amic acid solution was purchased from Sigma Aldrich (CAS: 25038-81-7, St. Louis, MI, USA). The PAA solution in N-methyl-2-pyrrolidone, NMP (CAS: 872-50-4, Vekton, Saint Petersburg, Russia) for R-BAPS was synthesized in the Institute of Macromolecular Compounds of Russian Academy of Sciences (IMC RAS). The diamine BAPS (CAS: 13080-89-2) was provided by Sisco Research Laboratories Pvt. Ltd., Navi Mumbai, India. The dianhydride R was purchased from Tech. Chim. Prom. Ltd., Yaroslavl, Russia.

For the hydrothermal synthesis of zirconia nanoparticles from inorganic precursors, ZrOCl_2_·8H_2_O (98.5%, CAS: 7699-43-6, Neva-Reaktiv, St. Petersburg, Russia) was used as a precursor and NH_4_OH solution (25%, CAS: 1336-21-6, Vekton, St. Petersburg, Russia) acted as a precipitator. To form nanoparticles of identical composition from organometallic compound, zirconium acetylacetonate powder (97%, CAS: 17501-44-9, Vekton, St. Petersburg, Russia) was mixed with toluene (99.5%, CAS: 108-88-3, Vekton, St. Petersburg, Russia), chosen as the reaction medium.

Toluene was used to provide supercritical conditions in the synthesis of TiO_2_ nanoparticles with anatase structure from titanium butoxide (purum, 97.0%, CAS: 5593-70-4, Sigma Aldrich, St. Louis, MI, USA) under the solvothermal treatment.

CNF (so-called vapor-grown carbon fibers, CAS: 308063-67-4) and CNT (CAS: 308068-56-6) were purchased from Sigma Aldrich (St. Louis, MO, USA) and annealed at 105 °C (for 1 h) before use to remove adsorbed moisture. 

**Figure 1:**
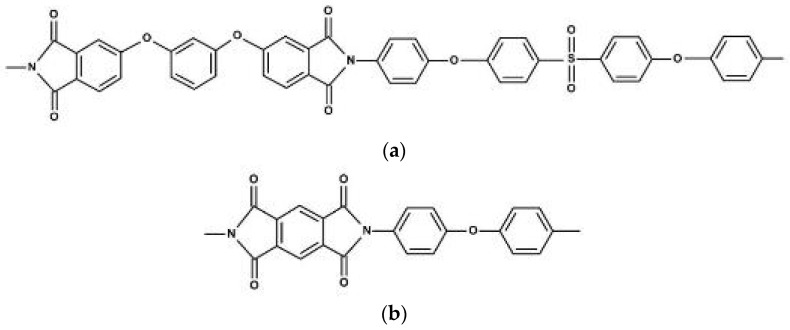
Structural formulas of the elementary units of the PIs: (**a**) R-BAPS; (**b**) PMDA-ODA.

Structural formulas of the elementary units of PIs used in this work are shown in Figure 1.

### 2.2. Synthesis and Treatment of Nanoparticles

The synthesis of ZrO_2_ nanoparticles with a high content of OH-groups on the surface was carried out in two stages. First, 25% ammonium hydroxide was added dropwise to a 1 M solution of ZrOCl_2_·8H_2_O until a white cheesy precipitate formed at pH = 14. Then, the ZrO(OH)_2_ precipitate was repeatedly washed with distilled water until a negative reaction to chloride ions occurred and it dried to a constant weight at 100 °C in a furnace. Next, 0.5 g of zirconium oxyhydroxide powder, ground in a mortar to a powdery state, was placed in a Teflon cell and was poured with 14.5 mL of distilled water. Then, the steel autoclave was hermetically sealed, heated at a rate of 5 °C/min, and kept at 250 °C for 4 h. The ZrO_2_ nanoparticles formed were removed from the autoclave, dried at 100 °C and cooled to room temperature naturally. The powder was characterized using a set of methods of physicochemical analysis [62]. The presence of the hydroxyl groups on the surface of the nanoparticles was confirmed by FTIR spectroscopy (Figure S1). These nanoparticles were designated ZrO_2(OH)_.

ZrO_2_ nanoparticles with a more hydrophobic surface were formed under solvothermal conditions. A suspension of zirconium acetylacetonate in toluene was subjected to the isothermal exposure for 72 h at a temperature of 250 °C and a pressure of 70 MPa. The formed particles were then repeatedly washed with ethanol, dried to a constant weight, and annealed in air at 500 °C for 2 h, or at 800 °C for 10 min, depending on the required crystallite size [63]. The nanoparticles of crystallite sizes 8 and 18 nm (according to X-ray phase analysis) were obtained and denoted by ZrO_2_(8nm) and ZrO_2_(18nm), correspondingly.

Solvothermal treatment under the same conditions was performed on a mixture of titanium butoxide with toluene in autoclaves. TiO_2_ nanoparticles with the anatase structure were formed [64], and annealed at 500 °C for 2 h.

In order to prevent the aggregation of CNT and improve their compatibility with the PI matrices [61,65] we modified their surfaces, exploiting the following technique: CNT were treated with boiling concentrated nitric acid for 36 h in a ratio CNT(g)/nitric acid(mL) 1:40. The powder obtained was washed with distilled water from nitric acid residues with the use of a centrifuge until pH = 7 was reached. The nanoparticles functionalized were then freeze-dried and denoted by 40% CNT_f_. The FTIR spectra of the pristine and processed CNT were registered to confirm the functionalization (Figure S2).

### 2.3. Synthesis of R-BAPS Prepolymer

A 15% solution of PAA based on diamine BAPS and dianhydride R was obtained by the polycondensation method. First, the diamine was dissolved in NMP. Then, an equimolar amount of dianhydride was gradually added to the solution under continuous stirring and cooling in an ice bath. The resulting solution was stirred for 4 h at room temperature in argon flow. The formed prepolymer was filtered and degassed. Prior to the synthesis, all monomers were dried for 24 h at temperatures 20 °C below their melting points. NMP was distilled under vacuum just before using in the synthesis of R-BAPS polyimide.

### 2.4. Preparation of Pristine and Nanocomposite Films

All the PI-based compositions (both with single and binary nanofillers) were prepared employing a standard solution technique [66,67]. The proper amounts of the nanoparticles, either MO or nanocarbon or their mixture, were sonicated in NMP for 1 h and then blended with the corresponding PAA solutions. The sizes and concentrations of the MO nanoparticles were chosen based on our previous results to provide the diversity of thermal and mechanical behavior of PIs filled with these nanospecies [58]. The choice of the amounts of the nanocarbon species was also based on our previous tests (results are not presented) ensuring optimal properties of the single-filler nanocomposites. The nanocomposite solutions obtained were stirred for 24 h to form a quasi-homogeneous system. These compositions as well as the host PAA solutions were cast on glass supports and dried for 4 h at 80 °C. A gradual heating up to either 365 °C (for PMDA-ODA-based samples) or 300 °C (for R-BAPS-based samples) was performed at a rate of 3 °C/min in order to prevent bubbling of the solvent. The final curing at these temperatures was carried out for 30 min. A list of the samples obtained is provided in Table 1.

### 2.5. Characterization Techniques

We investigated the structural and morphological features of the pristine polymer as well as nanocomposite samples employing a number of techniques.

Optical images of the surfaces of the nanocomposite samples were obtained, employing an ADF PRO20 digital microscopy camera (ADF Optics CO, Ltd., Hangzhou, China).

Scanning electron microscopy (SEM) was carried out on the cryo-cleavages of the films using a SUPRA-55VP microscope (Carl Zeiss, Oberkochen, Germany) equipped with a secondary electron detector. The specimens were glued on the microscope holders and covered with a thin layer of platinum. 

Atomic force microscopy (AFM) data were obtained using an SPM-9700HT scanning probe microscope (Shimadzu, Kyoto, Japan). The AFM images were captured in air at room temperature. The setup operated in a tapping mode using NSG-10 Silicon tips with curvature radius 5 nm. The images of 1024 × 1024 points were obtained.

FTIR spectra of unfilled and nanocomposite films were recorded on a Vertex 70 IR Fourier spectrometer (Bruker, Billerica, MA, USA) with the ATR (Attenuated Total Reflection) reflector (Pike Technologies, WI, USA) at room temperature in the range of 4000–400 cm^–1^ (number of scans 30) with a ZnSe working element. When registering the ATR spectra, a correction was made that includes the penetration depth depending on the wavelength. The equipment was also employed to confirm the presence of functional groups on the surfaces of ZrO_2(OH)_ nanoparticles and CNT_f_.

An X-ray phase analysis of the samples was performed on a Rigaku SmartLab 3 diffractometer (Rigaku Corporation, Tokyo, Japan) with CuKα radiation. The diffraction (XRD) patterns were taken in the range of angles 2θ = 10–60° at a speed of 1°/min. The phase composition of the nanoparticles was determined with the use of PDWin 4.0 software (NPO “Burevestnik”, St. Petersburg, Russia) using the profile analysis of XRD patterns. The results of the analysis were compared with the ASTM database. The crystallite sizes were calculated from the broadening of the X-ray diffraction lines according to the Scherrer equation [68].

A thermogravimetric analysis (TGA) of the materials was conducted using a DTG-60 setup (Shimadzu, Kyoto, Japan). The samples were heated up to 600 °C at a rate of 5 °C/min in air flow (100 mL/min). The thermal stability index, τ_10_ (the temperature at which a polymer or a composite loses 10% of its initial weight because of thermal destruction) was determined using TGA curves.

A thermomechanical analysis (TMA) in extension mode was applied in order to investigate the behavior of the films under heating. A TMA 402 F1 Hyperion thermal Analyzer (NETZSCH, Selb, Germany) was used. The samples were heated at a rate of 5 °C/min in argon flow (70 mL/min). Glass transition temperatures T_g_ were determined from TMA curves. A standard 0.5 MPa loading was applied to the PMDA-ODA-based samples. In the case of the films based on the flexible R-BAPS, the external stress was 25 kPa, since due to the steep transition to a plastic state, this polymer stretched far beyond the deformation range registered by the setup. 

Mechanical characteristics of the nanocomposite and reference PI films were studied with the use of an AGS-X 5kN (Shimadzu, Kyoto, Japan) setup. Mechanical tests were performed in a uniaxial extension mode at room temperature. Strip-like samples 2 × 20 mm in size were stretched at a rate of 10 mm/min. The Young’s modulus E, yield stress σ_y_, break stress σ_b_, and the ultimate deformation ε_b_ for each sample were determined. For each composition 8–10 strips were tested, and the average values were calculated.

## 3. Results and Discussion

### 3.1. Structure and Morphology

**Figure 2:**
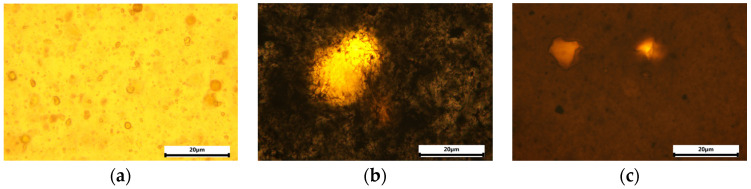
Optical images of R-BAPS-based nanocomposites with (**a**) single TiO_2_ nanofiller and binary mixtures of (**b**) TiO_2_/CNF and (**c**) TiO_2_/CNT_f_.

The optical images of the surfaces of the nanocomposites we prepared are displayed in Figure 2. It can be seen that the clustering of the MO nanoparticles took place, because the micron-sized aggregates were registered. This indicates the certain heterogenization of the material. However, the results of the study of samples by SEM, as well as strong effects of the nanofiller on the functional characteristics of the nanocomposites we observed, convincingly indicate that a large part of the nanofiller is uniformly distributed within the PI matrix.

**Figure 3:**
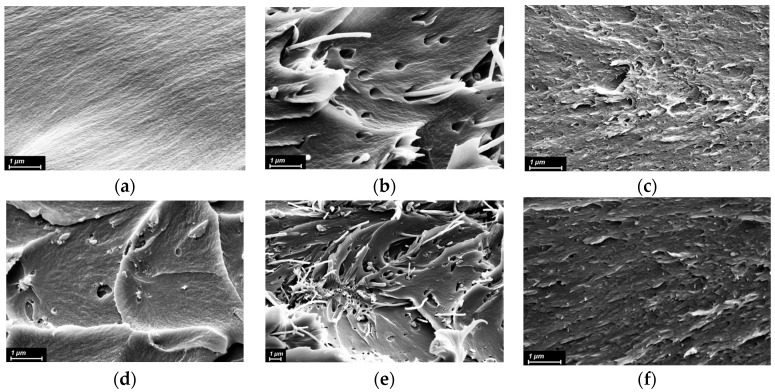
SEM images of (**a**) pristine R-BAPS matrix; (**b**) R-BAPS-based nanocomposite with CNF; (**c**) R-BAPS-based nanocomposite with CNT_f_; (**d**) R-BAPS-based nanocomposite with TiO_2_; (**e**) R-BAPS-based nanocomposite with TiO_2_/CNF mixture; (**f**) R-BAPS-based nanocomposite with TiO_2_/CNT_f_ mixture.

**Figure 4:**
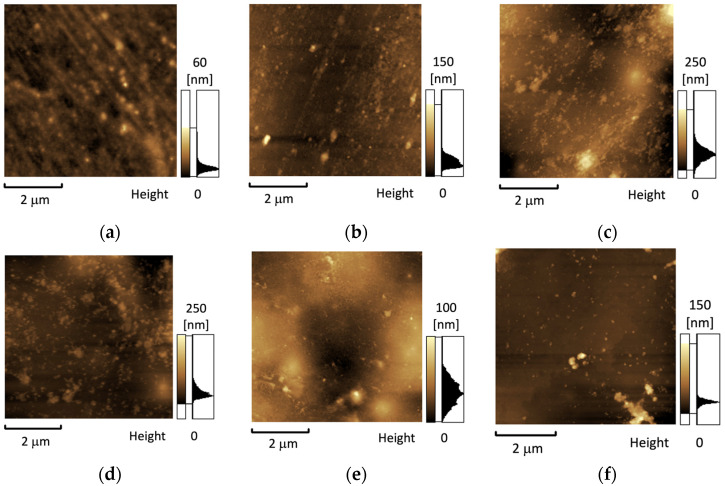
AFM images of (**a**) pristine R-BAPS matrix; (**b**) R-BAPS/TiO_2_ nanocomposite; (**c**) R-BAPS/CNF nanocomposite; (**d**) R-BAPS/TiO_2_/CNF nanocomposite; (**e**) R-BAPS/CNT_f_ nanocomposite; (**f**) R-BAPS/TiO_2_/CNT_f_ nanocomposite.

To investigate the interfacial interactions between the PI matrix and nanofillers and to assess the distribution of the nanoparticles within the matrix, we analyzed the SEM images (Figure 3) of the fractured surfaces and AFM images (Figure 4) of the surfaces of the films. The signs of plastic deformation were found on the fractured surfaces of all the nanocomposite samples (Figure 3b–f). It is apparent from Figure 3b that CNF had almost no adhesion to the matrix, providing a host of cavities inside the latter. In contrast, a cryo-cleavage of a CNT_f_-containing nanocomposite seemed uniform enough (Figure 3c). In the PI/TiO_2_ nanocomposite film, the nanoparticles were observed to aggregate, but they have good adhesion to the polymer matrix and are well-embedded in it (Figure 3d). Considering Figure 3e,f, one should conclude that the morphology of the nanocomposites filled with binary fillers is determined mostly by the carbon nanospecies, rather than by MO nanoparticles. Nonetheless, the sample with TiO_2_/CNF nanofiller has fewer voids compared with the single CNF-filled nanocomposite (Figure 3b,e). This results from the adhesion of the MO nanospecies to the matrix.

Figure 4 shows AFM images of the surfaces of the films. Analysis of AFM images of initial R-BAPS film (Figure 4a) shows a smooth morphology with roughness R_q_ = 3.8 nm (root mean square (RMS) roughness). We previously observed that morphology of PI-based nanocomposites is dependent on the type of MO [58]. The addition of TiO_2_ to R-BAPS led to the increase in roughness up to R_q_ = 6.3 nm (Figure 4b). At the same time, in the case of CNF (Figure 4c) as a filler, the surface roughness significantly increased up to R_q_ = 32.5 nm. This can be explained by rather large sizes of CNF. The nanocomposite filled with a binary mixture of TiO_2_/CNF showed smoother morphology with R_q_ = 24.6 nm which was likely caused by denser packing of the material containing TiO_2_ nanoparticles. This corresponds well with SEM images (Figure 3b,e). Moreover, the MO nanoparticles on the film surface can evidently decrease the difference between the heights on the topography maps. The values of surface roughness of CNT_f_-containing nanocomposites were quite close, R_q_ = 13.8 nm and 12.8 nm for R-BAPS/CNT_f_ and R-BAPS/TiO_2_/CNT_f_ compositions, respectively (Figure 4e,f).

**Figure 5:**
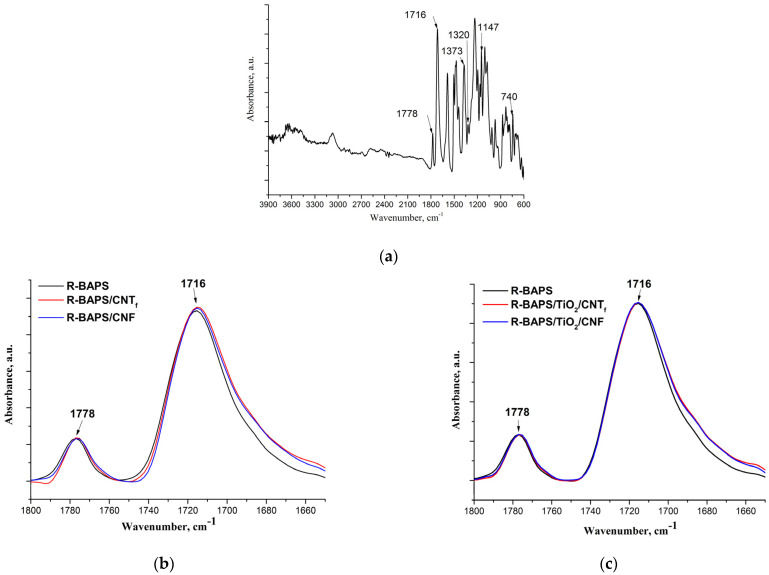

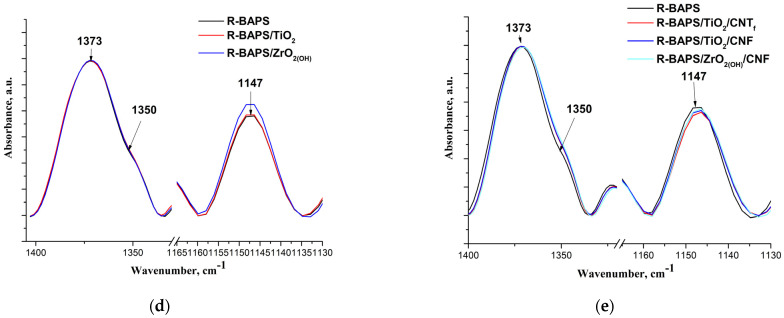
FTIR spectra of film samples’ (**a**) initial R-BAPS, (**b**–**e**) fragments of FTIR spectra of the initial R-BAPS, and nanocomposites with various types of nanoparticles.

The FTIR spectrum of the pristine R-BAPS is shown in Figure 5a. The characteristic peaks of the imide rings at 1778 cm^−1^, 1716 cm^−1^ (symmetric and antisymmetric C=O stretching vibrations), 1373 cm^−1^ (C-N stretching vibrations) and 740 cm^−1^ (deformation C=O vibrations) were registered [67]. The peaks at 1350 cm^−1^ (a shoulder near 1373 cm^−1^) and 1147 cm^−1^ were ascribed to SO_2_-group vibrations. The spectra of R-BAPS-based nanocomposites (Figure 5b–e) also exhibited distinctive imide ring absorption peaks. This indicates the completion of the PI imidization process not affected by the presence of the fillers. However, some changes in the peak positions were observed. When CNT_f_ and CNF were inserted in the R-BAPS matrix, a certain shift to the low-frequency region and widening of the bands corresponding to symmetric and antisymmetric C=O stretching vibrations of the imide cycle took place (Figure 5b), which may be attributed to the development of a network of hydrogen bonding. The shift of the same peaks in the R-BAPS/MO nanocomposites was rather small, which is likely due to the low concentration of the nanoparticles. Filling R-BAPS with binary MO/nanocarbon mixtures led to the same changes in the shapes of the bands and peak positions of C=O stretching vibrations as in the case of filling this PI with CNT_f_ and CNF nanospecies individually (Figure 5c). The C-N and SO_2_-group stretching vibrations were also affected by the presence of the nanofillers. It is evident that ZrO_2(OH)_ nanoparticles augmented the intensity of the 1147 cm^−1^ band, the effect of TiO_2_ on this band being very small (Figure 5d). Some changes in the intensities of the bands 1350 cm^−1^ and 1147 cm^−1^ along with a slight shift of the 1373 cm^−1^ peak to the low-frequency region were observed upon filling the R-BAPS with binary mixtures of MOs with nanocarbon (Figure 5e). Overall, all the nanofillers are proved to affect the matrix due to the formation intermolecular bonds between the PIs’ macromolecules and the surfaces of the nanoparticles. The nanosized TiO_2_, CNT_f_, and CNF interact both with the imide cycle (this presumably results in the changes in packing of the macromolecules) and with SO_2_-groups, while ZrO_2(OH)_ nanoparticles connect predominantly with SO_2_-groups.

**Figure 6:**
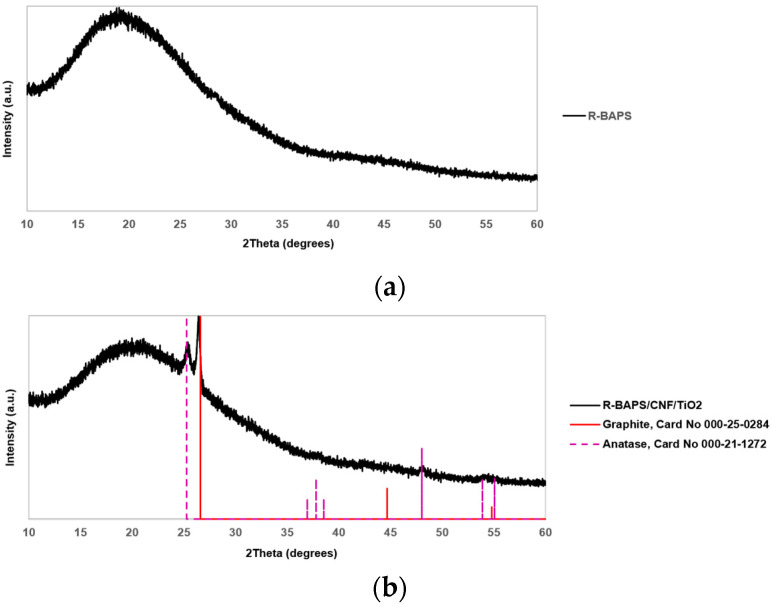

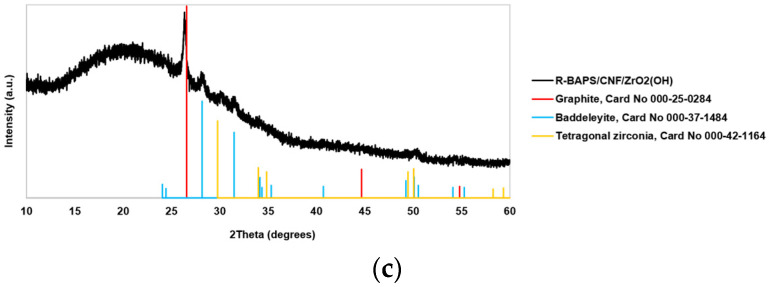
XRD patterns of (**a**) R-BAPS; (**b**) R-BAPS/CNF/TiO_2_; and (**c**) R-BAPS/CNF/ZrO_2(OH)_ nanocomposites.

Figure 6 shows the XRD patterns of the R-BAPS-based samples. A wide halo in the angle region of 10–35° reveals an amorphous structure of R-BAPS. The position and shape of this signal were unchanged upon the insertion of the nanofillers in the PI matrix implying that the polymer structure remains amorphous in the nanocomposites. Reflection at 26.4° corresponding to the crystallographic plane (200) of crystalline graphite (card No. 000-25-0284) was registered. An additional reflection characteristic of the crystallographic plane (101) of the anatase phase of TiO_2_ was fixed at 25.3° (card No. 000-21-1272) in R-BAPS/TiO_2_/CNF (Figure 6b). The XRD pattern of the R-BAPS sample containing CNF and ZrO_2(OH)_ (Figure 6c), along with the graphite reflection, contains peaks of baddeleyite at 28.2° and 31.6°, as well as a reflection of tetragonal zirconia at 30.6°. The average sizes of crystallites of oxide nanofillers from the reflections identified in film materials were estimated using the Scherrer formula and are equal to 20 nm for ZrO_2(OH)_, and 14 nm for TiO_2_.

### 3.2. Mechanical Properties

**Figure 7:**
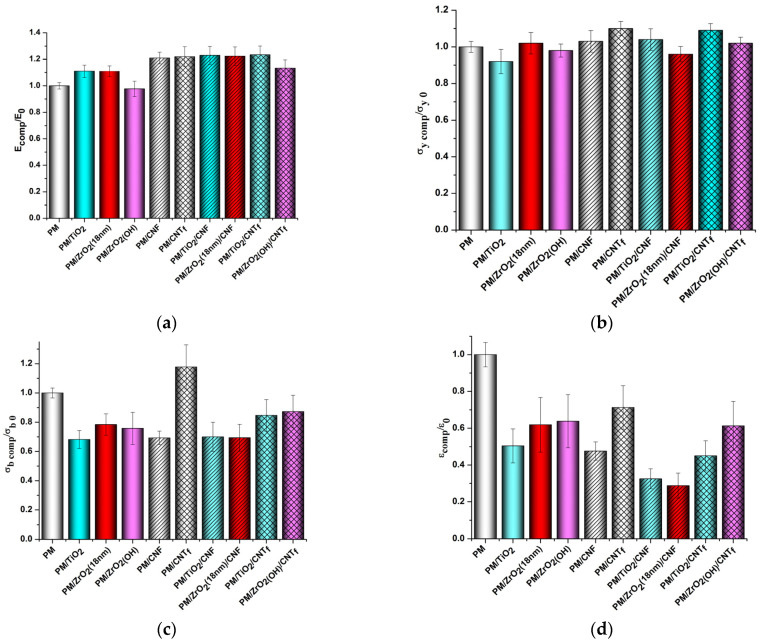
The effect of nanoparticles on the mechanical characteristics of PMDA-ODA (abbreviated as “PM” in the X-axis caption). (**a**) Young’s modulus, (**b**) yield stress, (**c**) break stress, (**d**) elongation at break.

**Figure 8:**
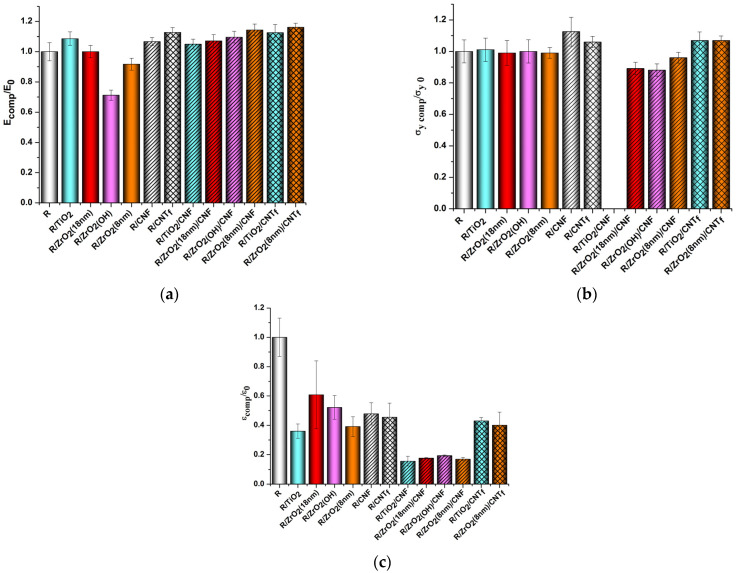
The effect of nanoparticles on the mechanical characteristics of R-BAPS (abbreviated as “R” in the X-axis caption). (**a**) Young’s modulus, (**b**) yield stress, (**c**) elongation at break.

Figure 7 and Figure 8 demonstrate the effect of various nanofillers on the mechanical properties of PMDA-ODA- and R-BAPS-based samples. Almost in all the nanocomposite samples, both MO and carbon nanoparticles are seen to augment the rigidity of the PIs. The positive influence of titania and zirconia nanoparticles is usually attributed to a certain degree of linking between PI macrochains and the surfaces of the nanoparticles via hydrogen bonding. One should mention that this effect depends strongly on the size, concentration, and surface functionality of MO nanospecies [50]. For instance, the smallest nanoparticles have the highest surface energy and a tendency to aggregate. In this case, the aggregates become stress concentrators and loosen the polymer structure (compare R-BAPS/ZrO_2_(18nm) with R-BAPS/ZrO_2_(8nm) samples in Figure 8). Mechanical characteristics of PI/MO nanocomposites are also affected by the surface functionality of the nanoparticles. The stiffness values for PI/ZrO_2(OH)_ nanocomposites were observed to be lower than those for the pristine PIs. This implies a negative effect of surface OH-groups on the structure of the PI material. Figure S3a,c shows that ZrO_2(OH)_ nanoparticles form large aggregates dispersed ununiformly within the matrix, a lot of cavities being also formed. This changes the morphology and topography of the PI, substantially increasing its roughness. The more palpable reinforcing effect of the nanocarbon species is ensured by the formation of a strong network consisting of 1D particles. This network is capable of taking over the external mechanical load from a polymer matrix [69,70]. Moreover, with CNT_f_ being preliminarily functionalized, chemical interactions between the PI macromolecules and these nanoparticles are provided, imparting even better properties to such nanocomposites [61].

The addition of the binary nanofillers to the PIs led to an increase in their rigidity, the Young’s moduli turning out to become higher than those of the PIs filled with only MO nanoparticles. For instance, the elasticity modulus of the PMDA-ODA/TiO_2_/CNF and PMDA-ODA/TiO_2_/CNT_f_ increased by 11% regarding the corresponding PI/TiO_2_ composite. It is worth noting that the difference between these values was more sensible if PI/MO nanocomposites had originally poor properties, e.g., PI/ZrO_2(OH)_ and PI/ZrO_2_(8nm) samples vs. PI/ZrO_2(OH)_/nanocarbon and PI/ZrO_2_(8nm)/nanocarbon composites. For example, the Young’s modulus of the R-BAPS/ZrO_2(OH)_/CNF sample was 54% higher than that of the R-BAPS/ZrO_2(OH)_ nanocomposite (Figure 8a). It is the nanocarbon networks that determine the stiffness and mechanical strength of the nanocomposites, with the MO nanoparticles being embedded and bridged into the network by 1D carbon nanospecies. The differences in the morphology and topography of the nanocomposites containing single ZrO_2(OH)_ filler and binary ZrO_2(OH)_/CNF can be seen in Figure S3.

There are two groups of mechanical characteristics reflecting the behavior of the film polymer material at different stages of the deformation process. Each of the groups is affected by different factors. For instance, the values of E and σ_y_ are determined predominantly by the strength of the system of intermolecular bonds in the material. On the other hand, break stress and elongation at break reflect the features of the material’s morphology, such as structural heterogeneity, and the presence and concentration of local internal defects, which occur at the phase boundaries during the processing of the nanocomposite. The differences in the magnitude of the characteristics from the second group (σ_b_ and ε_b_) show exactly the differences in the degree of microheterogeneity of the structure of the nanocomposite materials formed by adding various nanoparticles into the polyimide matrix. However, one should mention that from a practical viewpoint, such characteristics as E and σ_y_ are of great importance since generally PI materials are exploited exactly in the range of small deformations where the behavior of a material is characterized by these two parameters.

### 3.3. Thermal Properties

**Figure 9:**
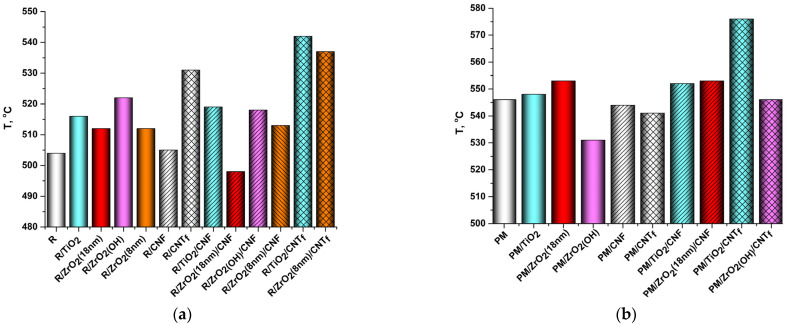
Thermal stability indices, τ10, of (**a**) R-BAPS(R)-based and (**b**) PMDA-ODA(PM)-based materials.

The thermal stability indices τ_10_ of the pristine PIs and PI-based nanocomposites are shown in Figure 9. All the materials are proven to possess excellent thermal stability. The thermal degradation of nanoparticles per se (both metal oxides and carbon nanoparticles) begins at temperatures significantly higher than the onset temperatures of thermal degradation of the matrix polymers studied in the work. Thus, the influence of nanosized fillers on the thermal stability indices of the nanocomposite materials determined in the work is ensured by their impact on the system of intermolecular bonds and the morphology of the material (in particular, on the structure of the interfaces between the polymer and fillers). It is obvious from Figure 9 that nanoparticles affect the thermal stability of the PIs variously. As we already discussed in [54,58] the effect is determined by a number of factors including the type, size, surface functionality, and concentration of nanofiller. Considering MO nanoparticles, their positive influence on the thermal stability of PIs containing SO_2_-groups (R-BAPS) can be caused by two reasons: The first one is presumed to be the ability of these nanospecies to take part in chemical reactions with atmospheric oxygen at elevated temperatures providing additional links between positively charged S atom in PI radicals and the surface of nanoparticles bearing negative charge due to active oxygen species (AOS) formed. These links can slow down the degradation of a nanocomposite film in a certain region of temperatures (Figure 9a). However, the AOS may well have a detrimental effect on a PI matrix (PMDA-ODA) with no sulfonyl groups catalyzing its thermo-oxidative decomposition (Figure 9b), e.g., in PMDA-ODA/3%ZrO_2(OH)_ nanocomposite [23,58]. On the other hand, improved mechanical properties of PI/MO nanocomposites discussed above imply good interfacial interactions between the matrix and filler. These interactions can provide a physical barrier, retarding the out-diffusion of volatile products during thermal degradation of the nanocomposites. The effect of CNF on the τ_10_ values of PIs is generally less pronounced, at least at the concentrations provided. This agrees well with the data we obtained earlier [54]. The difference in thermal behavior of CNF- and CNT_f_-containing nanocomposites can be attributed to the profound contrast in their morphology (Figure 3b,c). The denser and more homogeneous structure of R-BAPS/CNT_f_ nanocomposite is evidently responsible for its high thermal stability.

The introduction of binary nanofillers into the PI matrices causes the enhancement of their thermal stability, which becomes predominantly even higher than those of the corresponding single-component nanocomposites. The most pronounced effect was observed in the nanocomposites filled with the TiO_2_/CNT_f_ combination, whose τ_10_ were augmented by more than 30 °C compared with these values for the pristine R-BAPS and PMDA-ODA matrices.

Putting together SEM images (Figure 3) and the data on mechanical characteristics (Figure 8), the synergism in R-BAPS-based nanocomposites with binary nanofillers is supposed to stem from the simultaneous change in the structure of the matrix (provided by carbon nanospecies) and specific interactions of the MO nanoparticles with SO_2_-containing macrochains. For instance, comparing Figure 3d,e one can notice that the morphology of a nanocomposite with mixed TiO_2_/CNT_f_ differs considerably from that of the sample with the single TiO_2_ nanofiller and is determined by the nanocarbon filler. It is seen from Figure 3b, that the R-BAPS/CNF sample has many caverns due to the presence of CNF. These caverns may well-facilitate the out-diffusion of the PI degradation products. Despite the R-BAPS/TiO_2_/CNF structure being loose, one can observe an increase in thermal stability of the nanocomposite, which is supposed to be ensured by titania. As for PMDA-ODA-based samples, the positive effect of the binary filler on their thermal stability obviously results primarily from the change in the structure of the materials due to the addition of the nanocarbon. Moreover, carbon nanospecies can likely cover the active surface of the MO nanoparticles (Figure 2b,c), thereby hindering their catalytic effect on thermo-oxidative destruction of PMDA-ODA.

Similar results were obtained in [54] for PMDA-ODA and another PI-bearing sulfonyl groups (DPhO-BAPS) filled with binary CeO_2_/nanocarbon mixtures. The synergistic effect of a mixture of carbon nanoparticles (GNPs) with inorganic salt (BN) on thermal stability of a PI was also registered in [52], but the concentration of the salt reached 30 wt.% so as to provide an increase in τ_5_ value less than 10 °C.

### 3.4. Thermomechanical Properties

**Figure 10:**
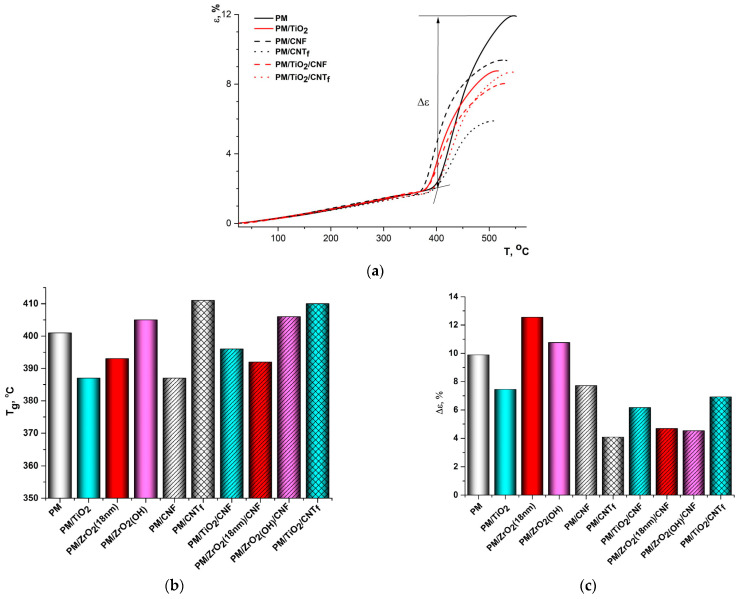
The effect of various nanofillers on the thermomechanical behavior of PMDA-ODA (abbreviated as “PM” in the X-axis caption). (**a**) TMA curves of PMDA-ODA-based samples, (**b**) the T_g_ values of PMDA-ODA-based samples, (**c**) the Δε values PMDA-ODA-based samples.

**Figure 11:**
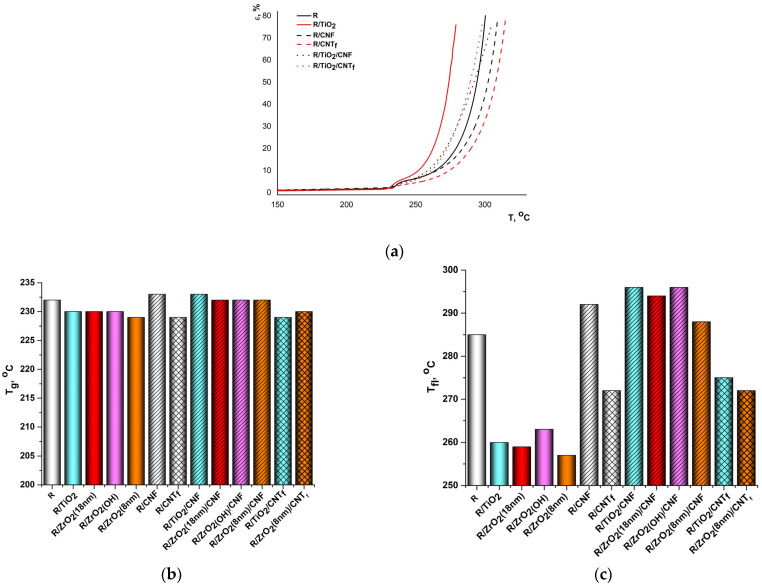
The effect of various nanofillers on the thermomechanical behavior of R-BAPS (abbreviated as “R” in the X-axis caption). (**a**) TMA curves of R-BAPS-based samples, (**b**) the T_g_ values of R-BAPS-based samples, (**c**) the T_fl_ values R-BAPS-based samples.

Figure 10a and Figure 11a illustrate TMA curves of a series of the PMDA-ODA- and R-BAPS-based samples. The nanofillers were demonstrated to affect T_g_ diversely (Figure 10b and Figure 11b). Almost all the MO nanoparticles generally decreased T_g_ of both the PIs. We discussed such an effect in detail in [58]. It is noteworthy that the influence of the MO nanospecies on T_g_ values is more substantial in PMDA-ODA samples. This can be explained by the fact that the flexible macromolecules of R-BAPS facilitate its segmental mobility at a temperature T_g_ lower than that of PMDA-ODA. Apparently, the structure of the polymer rather than the nanofiller plays a decisive role in the behavior of R-BAPS-based nanocomposites in the vicinity of the T_g_. It is also evident from Figure 10b that two types of nanoparticles augment the T_g_ of PMDA-ODA, namely ZrO_2(OH)_ and CNT_f_, whose surfaces bear functional groups. One can presume additional interactions between these groups and PMDA-ODA macromolecules retarding the segmental mobility of the polymer and increasing its T_g_.

It is seen from Figure 10a that the pristine PMDA-ODA was stretched quite strongly above the T_g_. When the temperature reached ~440 °C the deformation rate decreased, signaling an initial stage of the polymer thermal degradation accompanied with the formation of destruction crosslinks. The crosslinks are likely to stiffen the material in this temperature region [67,71]. To assess the rigidity of the samples above the T_g_, we determined a value of deformation in the temperature range between the T_g_ and a temperature of maximum deformation on the TMA curves, Δε (Figure 10c). Obviously, MO nanoparticles affect the Δε value of the nanocomposites diversely, the rigidity of the materials depending on the type, size, and concentration of the nanospecies. Generally, the increase in Δε value is observed when either the size or concentration of the MO nanoparticles is augmented, since more aggregates preventing polymer–polymer interactions are formed and more flexible behavior in PMDA-ODA-based nanocomposites is demonstrated after glass transition [58]. As concerns the nanocomposites filled with nanocarbon (either CNT_f_ or CNF), the Δε value was registered to drop against the host PMDA-ODA. The network of 1D carbon nanoparticles is presumed to contribute to the crosslinking at elevated temperatures and impede the motion of macromolecular segments [54]. Filling PMDA-ODA with binary MO/nanocarbon mixtures resulted in a rise in its stiffness compared with the corresponding nanocomposites with single MO nanofillers, implying that it is the nanocarbon network that ensures low Δε values of such materials.

Unlike PMDA-ODA, flexible R-BAPS stretches quite well even under little stress applied, exhibiting elastic behavior above the T_g_ followed by plastic deformations at higher temperatures. The mutual motion of the macrochains as a whole is eased significantly at temperatures ca. 50 °C higher than the T_g_. As a result, this PI begins flowing (temperature at which the flowing occurs is denoted as T_fl_) (Figure 11a). Such behavior is typical of the PIs containing several bridge groups [54,72]. Analyzing Figure 11c, one can observe that the MO nanospecies strongly affect T_fl_ decreasing this value by more than 25 °C. The quasi-spherical MO nanoparticles plausibly hinder the polymer–polymer interactions, facilitating a relative motion of the R-BAPS macromolecules. The same effect can be responsible for a drop in the T_fl_ in R-BAPS/CNT_f_ sample, since the polymer–nanoparticle (either MO or CNT_f_) crosslinked structures determining the stiffening of the corresponding nanocomposites at room temperature (Figure 8) may well be broken or at least become more flexible at higher temperatures. Despite the fact that CNF makes the polymer packing less dense (Figure 3b,e), which should promote the motion of the macrochains, the T_fl_ of the R-BAPS nanocomposites containing CNF is much higher than that of the pristine matrix. It appears that the mobility of the sample as a whole can be hindered because of a rigid network formed by large CNF incapable of “flowing” until a high temperature is reached. This network obviously determines the behavior of the R-BAPS-based nanocomposites filled with binary MO/CNF nanofillers, since their T_fl_ always surpasses the T_fl_ of the unfilled polymer.

## 4. Conclusions

In this research, we reported on the fabrication technique and comprehensive study of the thermal and mechanical properties of PI-based nanocomposites filled with binary mixtures of MO nanoparticles (either TiO_2_ or ZrO_2_) and nanocarbon (either CNF or CNT_f_). The structural and morphological features of the aforesaid materials were considered as well. The improvement of mechanical characteristics compared with single-filler (MO nanospecies) nanocomposites was demonstrated. It is the nanocarbon additive that is supposed to be responsible for the increase in stiffness (both below and above T_g_) and yield stress of the materials. On the other hand, the MO nanoparticles are likely to augment the thermal stability of the PI. As a result, novel materials with an extended set of enhanced properties were developed. The most pronounced synergistic effect of the constituents was registered in PIs filled with the binary TiO_2_/CNT_f_ mixture whose thermal stability (τ_10_ value) turned out to be higher than both single-filler nanocomposites, the films possessing excellent mechanical properties.

Filling PI with binary MO/carbon nanoadditives was proven to be an effective method of fabrication of nanocomposites to overcome the limitations of the single-filler systems. We believe that such a versatile approach to the design of new nanocomposite materials would be an ideal choice in high-end engineering applications.

## Figures and Tables

**Table 1:** A list of the nanocomposite samples based on polyimides.

Sample	PI	Nanofiller	Weight Content of Nanofiller, wt.%
PMDA-ODA	PMDA-ODA	-	0
PMDA-ODA/ZrO_2_(18nm)		ZrO_2_	5
PMDA-ODA/ZrO_2(OH)_			3
PMDA-ODA/TiO_2_		TiO_2_	3
PMDA-ODA/CNF		CNF	4.5
PMDA-ODA/CNT_f_		CNT_f_	3
PMDA-ODA/TiO_2_/CNF		TiO_2_/CNF	3/4.5
PMDA-ODA/ZrO_2_(18nm)/CNF		ZrO_2_(18nm)/CNF	5/4.5
PMDA-ODA/TiO_2_/CNT_f_		TiO_2_/CNT_f_	3/3
PMDA-ODA/ZrO_2(OH)_/**CNT_f_**		ZrO_2(OH)_/CNT_f_	3/3
			
R-BAPS	R-BAPS	-	0
R-BAPS/ZrO_2_(8nm)		ZrO_2_	3
R-BAPS/ZrO_2_(18nm)			3
R-BAPS/ZrO_2(OH)_			3
R-BAPS/TiO_2_		TiO_2_	3
R-BAPS/CNF		CNF	4.5
R-BAPS/CNT_f_		CNT_f_	3
R-BAPS/TiO_2_/CNF		TiO_2_/CNF	3/4.5
R-BAPS/ZrO_2_(18nm)/CNF		ZrO_2_(18nm)/CNF	3/4.5
R-BAPS/ZrO_2(OH)/_CNF		ZrO_2(OH)/_CNF	3/4.5
R-BAPS/TiO_2_/CNT_f_		TiO_2_/CNT_f_	3/3
R-BAPS/ZrO_2(OH)_/**CNT_f_**		ZrO_2(OH)_/CNT_f_	3/3

## Data Availability

Not applicable.

## Supplementary Materials

The following supporting information can be downloaded at: https://www.mdpi.com/article/10.3390/polym15102298/s1, Figure S1: IR spectra of initial ZrO_2_(8nm) (black) and ZrO_2(OH)_ (red) powders; Figure S2: FTIR spectra of the initial CNT (black) and CNT after functionalization, CNT_f_ (red); Figure S3: Scanning electron microscopy (SEM) images of (**a**) R-BAPS-based nanocomposite with ZrO_2(OH)_; (**b**) R-BAPS-based nanocomposite with ZrO_2(OH)_/CNF mixture. Atomic force microscopy (AFM) images of (**c**) R-BAPS-based nanocomposite with ZrO_2(OH)_; (**d**) R-BAPS-based nanocomposite with ZrO_2(OH)_/CNF mixture.
