# Supplementary material for: Synergistic Effect of Metal Oxide and Carbon Nanoparticles on the Thermal and Mechanical Properties of Polyimide Composite Films
Source: Polymers (Basel). 2023 May 13;15(10):2298. doi: 10.3390/polym15102298 (PMC10223102; doi:10.3390/polym15102298)
Supplement: Supplementary file 1 [file polymers-15-02298-s001.zip › polymers-2392533-supplementary.pdf]

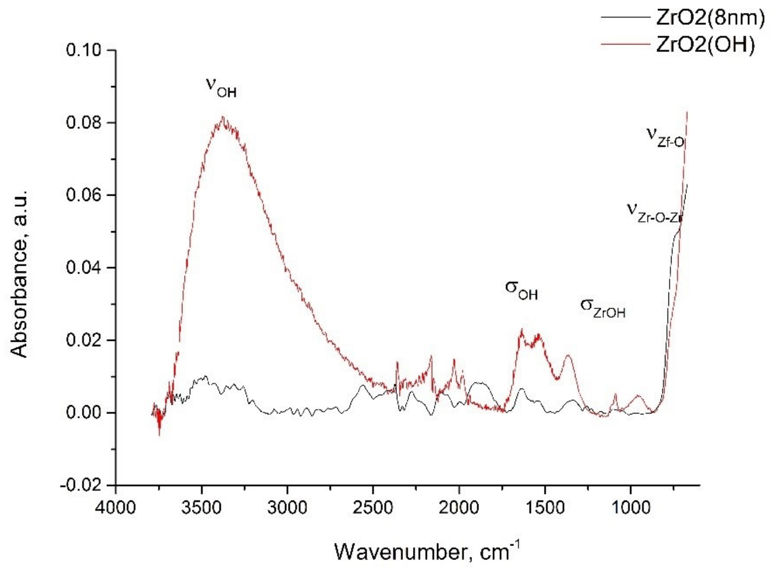

Figure S1. IR spectra of initial ZrO_2(8nm) (**black**) and $ZrO_{2(OH)}$ (**red**) powders.

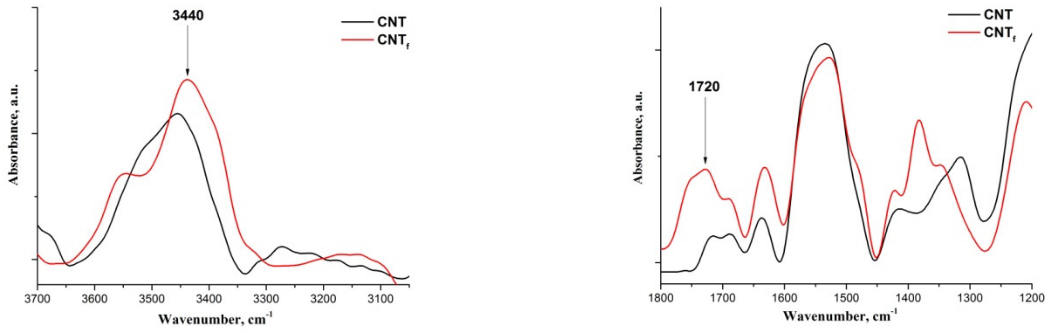

(a) (b)

Figure S2. IR spectra of the initial CNT (**black**) and CNT after functionalization, CNT$_f$ (**red**). Fragments of spectra in the wavenumbers regions (a) 3700-3050 and (b) 1800-1200 cm^{-1}.

The spectrum of CNT$_f$ exhibits peaks at 1720 cm^{-1} characteristic of a carbonyl group. The width and shape of the band reveal that the modification of CNT results in a number of C=O-containing compounds (saturated and unsaturated ketones, aromatic ketones, and acids) on the CNT$_f$ surface [1]. One should also mention a change in the position of the peak at 3400 cm^{-1}.

1. Gofman, I. V.; Abalov, I. V.; Vlasova, E.N.; Goikhman, M.J.; Zhang, B. Comparative Evaluation of Different Methods of Carboxylation of Carbon Nanotubes as a Modifier of Mechanical Properties of Heat-Resistant Polyimide Based Nanocomposites. *Fibre Chem.* **2015**, *47*, doi:10.1007/s10692-016-9671-z.

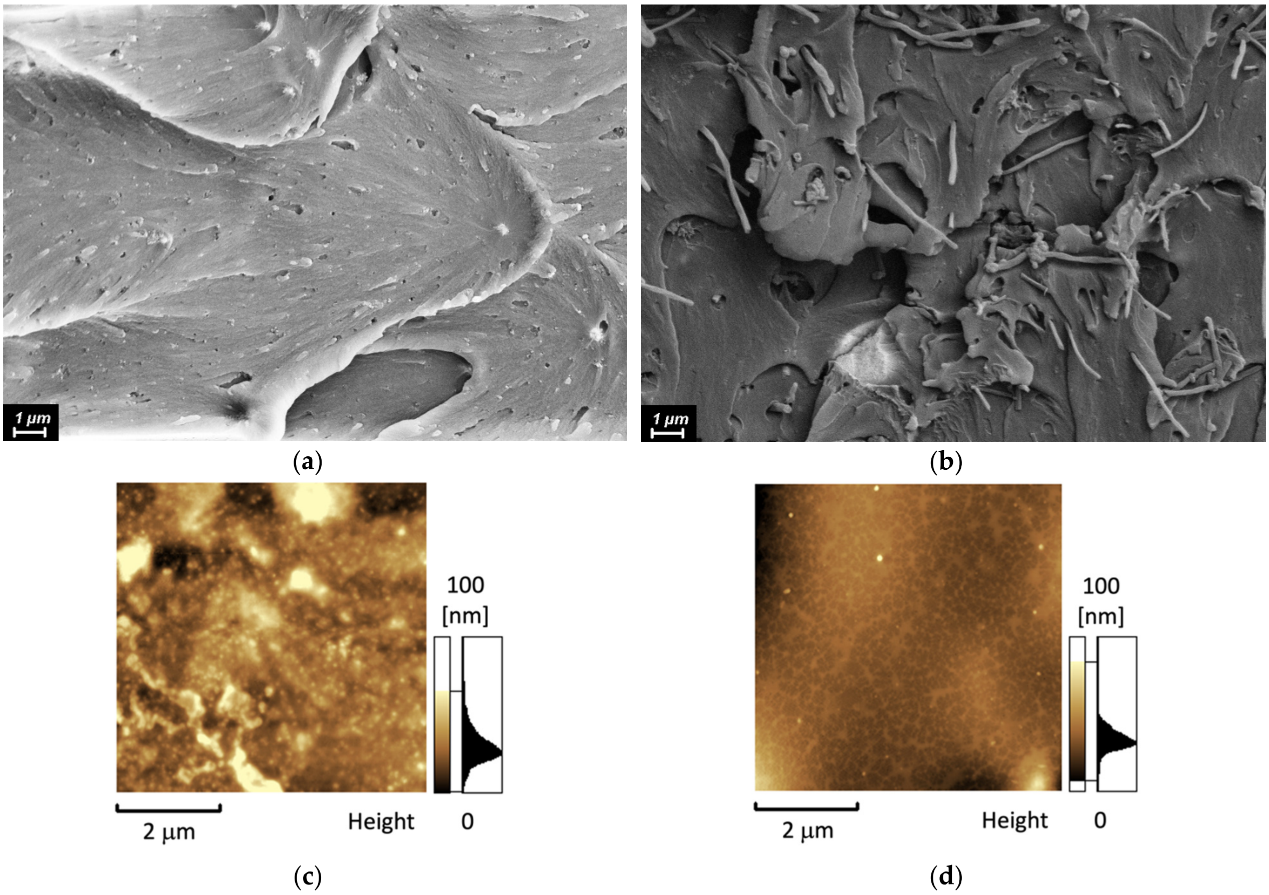

Figure S3. Scanning electron microscopy (SEM) images of (**a**) R-BAPS-based nanocomposite with $ZrO_{2(OH)}$; (**b**) R-BAPS-based nanocomposite with $ZrO_{2(OH)}$/CNF mixture. Atomic force microscopy (AFM) images of (**c**) R-BAPS-based nanocomposite with $ZrO_{2(OH)}$; (**d**) R-BAPS-based nanocomposite with $ZrO_{2(OH)}$/CNF mixture

*According to AFM, the $ZrO_{2(OH)}$-containing nanocomposite had clearly visible nodular morphology (R_q = 19.5 nm). We found that incorporating CNF into $ZrO_{2(OH)}$ nanocomposite film had dramatic effect on the surface topography and roughness, smoothening the surface of nanocomposite down to R_q = 10.1 nm.
